# Ultrasound-Triggered on Demand Lidocaine Release Relieves Postoperative Pain

**DOI:** 10.3389/fbioe.2022.925047

**Published:** 2022-07-11

**Authors:** Xiaohong Chen, Jianfeng Zhang, Yan Yu, Haoran Wang, Genshan Ma, Di Wang, Hanzhong Cao, Jianping Yang

**Affiliations:** ^1^ The Frist Affiliated Hospital of Soochow University, Suzhou, China; ^2^ Nantong Tumor Hospital, Tumor Hospital Affiliated to Nantong University, Nantong, China

**Keywords:** ultrasound-triggered drug release, postoperative pain, lidocaine, on-demand release, erythrocytes

## Abstract

Safe and non-invasive on-demand relief is a crucial and effective treatment for postoperative pain because it considers variable timing and intensity of anesthetics. Ultrasound modulation is a promising technique for this treatment because it allows convenient timed and non-invasive controlled drug release. Here, we created an ultrasound-triggered lidocaine (Lido) release platform using an amino acid hydrogel functioning as three-dimensional (3D) scaffold material (Lido-PPIX@ER hydrogel). It allows control of the timing, intensity and duration of lidocaine (Lido) to relieve postoperative pain. The hydrogel releases Lido due to the elevated reactive oxygen species (ROS) levels generated by PPIX under ultrasound triggering. The Lido-PPIX@ER hydrogel under individualized ultrasound triggering released lidocaine and provided effective analgesia for more than 72 h. The withdrawal threshold was higher than that in the control group at all time points measured. The hydrogel showed repeatable and adjustable ultrasound-triggered nerve blocks *in vivo*, the duration of which depended on the extent and intensity of insonation. On histopathology, no systemic effect or tissue reaction was observed in the ultrasound-triggered Lido-PPIX@ER hydrogel-treated group. The Lido-PPIX@ER hydrogel with individualized (highly variable) ultrasound triggering is a convenient and effective method that offers timed and spatiotemporally controlled Lido release to manage postoperative pain. This article presents the delivery system for a new effective strategy to reduce pain, remotely control pain, and offer timed and spatiotemporally controlled release of Lido to manage postoperative pain.

## Introduction

Postoperative pain management, particularly acute postoperative pain analgesia, remains a leading clinical problem (Gao et al., 2021; Joseph et al., 2021). Without personalized, adjustable, and convenient regional anesthesia, many patients experience intolerable pain. Studies have reported that 10%–50% of patients undergoing surgery experience postoperative pain lasting more than 1 month, and 2%–10% of these patients continue to experience moderate to severe chronic pain (Wang et al., 2020). Although many new devices for postoperative pain have been developed, inadequate postoperative analgesia occurs because the anesthetic cannot meet the on-demand requirements (Tobe et al., 2010; Wang et al., 2016; Rwei et al., 2017; Chen et al., 2020). Inadequate management of postoperative pain can lead to severe consequences, such as poor immediate postoperative effects, prolonged hospital stays, poor patient satisfaction, and increased burden on patients and health systems (Beaulieu, 2021; Joseph et al., 2021; Park et al., 2021). Thus, variability in the patients’ ability to modulate pain has clinical application in the control of postoperative pain.

Remotely triggered drug release systems have been developed that can meet the need to regulate pharmacological effects in a timely manner (Raoof et al., 2013; Park et al., 2019; Pedersen et al., 2020; Yao et al., 2021). Ultrasound is already commonly used in both hospital and family therapy for diagnostic and therapeutic practice (Tharkar et al., 2019; van Rooij et al., 2021). Ultrasound is non-invasive and can penetrate deep into tissues (Rwei et al., 2017). It can be applied in a focused manner to minimize the energy applied to the surrounding, non-targeted tissues. Many of the current ultrasound-triggerable drug release systems, such as micelles, liposomes, composites, and hybrid materials are responsive to the thermal and mechanical effects of ultrasound waves (Song et al., 2016; Singh et al., 2018; Jing et al., 2019; Kuls et al., 2020). To improve the effect of sonodynamic therapy, more sonosensitizers have been developed, such as protoporphyrin IX (PPIX) (Rwei et al., 2017). Ultrasound can induce sonochemistry—that is, the use of ultrasonic waves for chemical reactions in which sound sensitizers are activated by acoustic energy to generate reactive oxygen species (ROS). Remote-controlled ROS generation has been widely explored to trigger drug release (Xia et al., 2019).

Erythrocytes (ER), which can deliver various drugs, are attractive systems with adequate lifespans, large internal capacities, and good biocompatibility (Al-Achi and Greenwood, 1998; Hamidi et al., 2007; Favretto et al., 2013). Remote laser-controlled drug delivery systems, showing spatiotemporally controlled drug release, allow on-demand drug release without invasive injury (Wang et al., 2021). Photosensitisers generate reactive oxygen species (ROS) by laser irradiation, working as optical switches to be used in remote laser-controlled drug delivery systems (Strauss et al., 2017). Under remote stimulation, drugs are released into the bloodstream from the laser-regulated drug release system because of ROS generation, opening the ER phospholipid bilayer and triggering drug release. Once the remote stimulation disappears, ROS is no longer produced, and the generated ROS is scavenged by superoxide dismutase and catalase, leading to ROS exhaustion in the hydrogel (Xia et al., 2019). The pores on the ER membrane are then closed to terminate the release behavior.

**SCHEME 1:**
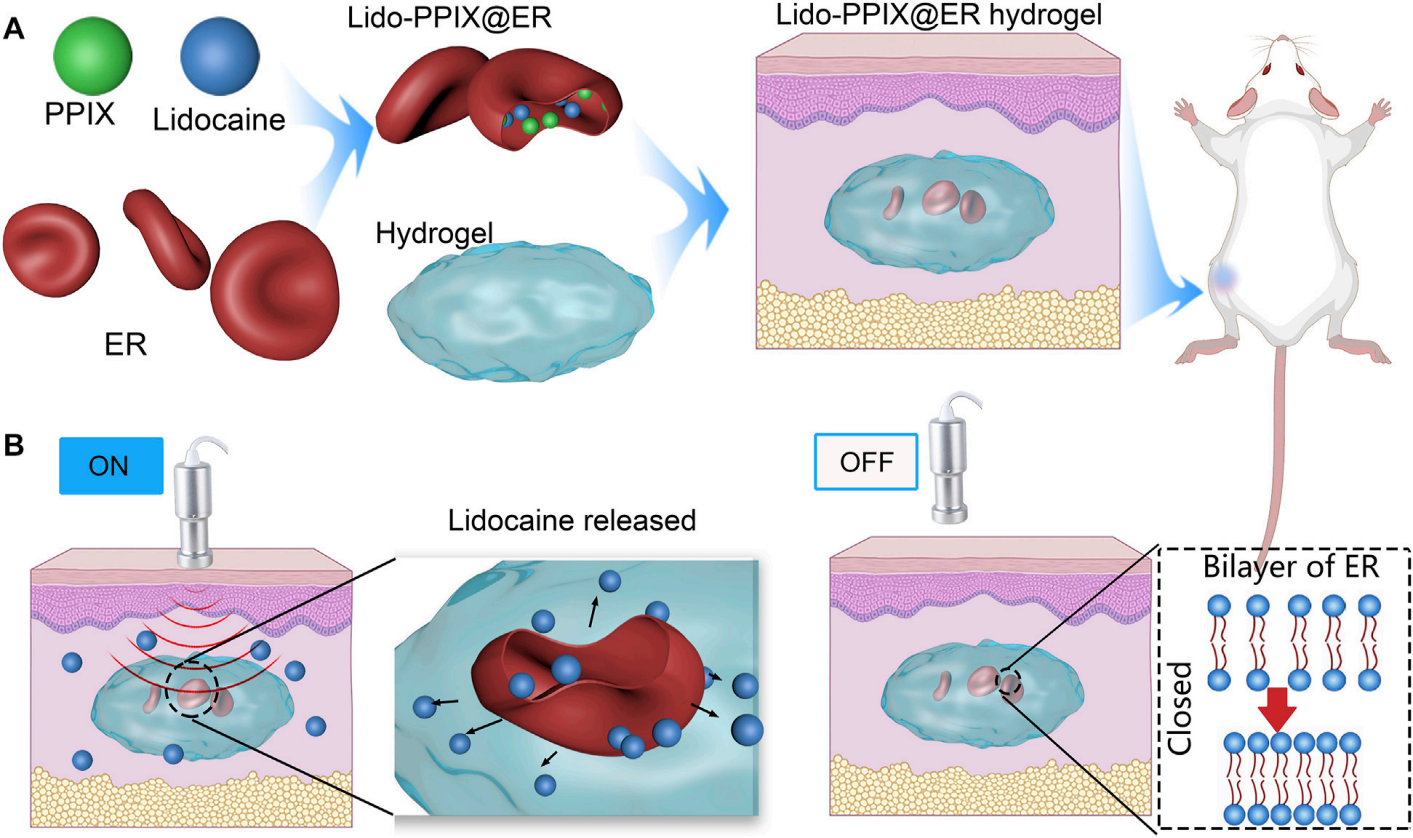
Schematic representation of erythrocyte-inspired and ultrasound-triggered Lido on demand release platform. **(A)** Formation of the Lido-PPIX@ER hydrogel in which ultrasound (0.3 W/cm^2^, 30 s) triggers the release of lidocaine (Lido). ERs (erythrocytes) were selected as the lidocaine and PPIX carrier. The amino acid hydrogel functions as a hydrogel 3D scaffold material, which offers increased subcutaneous ER stability. **(B)** A schematic representation showing the ultrasound-regulated Lido release from the Lido-PPIX@ER hydrogel. After surgery, following ultrasound irradiation (0.3 W/cm^2^), PPIX improves active oxygen production, promoting pore formation on the erythrocyte membrane, and allowing lidocaine release. Turning off the ultrasound leads to closure of the pores on the ER membrane, suppressing lidocaine release. The Lido-PPIX@ER hydrogel allows on-demand lidocaine release from the subcutaneous lidocaine reservoir by remote control.

Inspired by the characteristics of sonodynamic therapy and erythrocytes as drug carriers, an injectable erythrocyte-inspired, ultrasound-activated hydrogel (Lido-PPIX@ER hydrogel) was reported (Scheme 1). In this system, ERs are used as reservoirs for both Lido and PPIX (Scheme 1A), and the deformed hydrogel can be subcutaneously injected into the body and function as a 3D scaffold containing Lido- and PPIX-loaded ER to improve their subcutaneous stability. This Lido-PPIX@ER hydrogel releases Lido because of the elevated content of reactive oxygen species (ROS) generated by PPIX under ultrasound. Conversely, ROS are scavenged without ultrasound irradiation and stop Lido release from the Lido-PPIX@ER hydrogel (Scheme 1B). Thus, an ultrasound-triggered anesthetic release system with remote-controlled timing and intensity is obtained to regulate postoperative pain on demand. Collectively, we highlight the potential of this ER-enabled on-demand nerve block system, which can use off-the-shelf ultrasound clinical equipment and may ease the clinical transformation of on-demand drug delivery systems in postoperative pain management.

## Materials and Methods

### Materials and Animals

Lidocaine hydrochloride (5 ml: 0.1 g) was obtained from Tiancheng Pharmaceutical (Hebei, China). Protoporphyrin IX (PPIX) and dialysis bags (molecular cutoff, 0.5 kDa) were obtained from Bomei Biotechnology (Hefei, China). Fluorescein isothiocyanate lidocaine (FITC-Lido) was provided by Qiyue Biological Technology (Xi’an, China). Fmoc-Phe and Phe2 were purchased from Yuanye Biotechnology (Shanghai, China). *Lipase* from *Pseudomonas fluorescens* (PFL) was provided by Aladdin Industrial Corporation (Shanghai, China). An malondialdehyde (MDA) kit was obtained from Nanjing Jiancheng Bioengineering Institute (Nanjing, China). All other reagents were of reagent grade.

SPF Sprague–Dawley (SD) rats were purchased from the Experimental Animal Center of Nantong University. All the animals were housed at 24 ± 2°C, 40%–70% humidity, and a 12 h photoperiod. The procedures used in our study were approved by the Committee of the Animal Research of Nantong University (20210421-072), and followed the guidelines the Use of Animals in Research of the International Association for the Study of Pain and the directive of the European Parliament (2010/63/EU).

### Preparation of the Lido-PPIX@ER hydrogel

Fresh blood was collected from SD rats using an appropriate anticoagulant (heparin). ERs were normally centrifuged at 4°C and then washed with 10–15 ml of PBS solution at least three times. The hypotonic dialysis method was adopted to prepare the Lido-PPIX@ER. Briefly, PPIX was dissolved in 1 ml of dimethyl sulfoxide (DMSO). Next, lidocaine hydrochloride and PPIX were added to the ER suspension. The mixture was subjected to dialysis (molecular cut off, 0.5 kDa) before immersion in hypotonic buffer (72 mOsm/kg, pH 8) at 4°C for a set time and resealing buffer (550 mOsm/kg, pH 8) for 30 min at 37°C. Finally, free lidocaine hydrochloride and PPIX were removed using repeated centrifugation.

Hypotonic buffer was used to prepare Lido-PPIX@ER, comprising 15 mM NaH_2_PO_4_·2H_2_O, 15 mM NaHCO_3_, 2 mM ATP, 3 mM glutathione, 20 mM glucose, and 5 mM NaCl in 100 ml of distilled water, and the resealing buffer included 250 mM NaCl, 12.5 mM glucose, 12.5 mM sodium pyruvate, 12.5 mM inosine, 12.5 mM NaH_2_PO_4_·2H_2_O, 0.63 mM adenine, and 550 mOsm/kg in 100 ml of distilled water (Millán et al., 2004).

The deformable peptide hydrogel was prepared as described previously (Chronopoulou et al., 2012). Briefly, the substrates Fmoc-Phe and Phe2 were suspended in 420 µL of 0.5 M NaOH. The final concentrations of the Fmoc-Phe derivative and Phe2 were 11.3 and 8.5 mg/ml, respectively. Next, 500 µL of Lido-PPIX@ER, 0.1 M catalase, and SOD were added and dispersed in the suspension using a magnetic stirrer. The pH (7.0) was adjusted using 0.1 M HCl, and a final volume of 3.5 ml was obtained. Next, 100 µL of 50 mg/ml of lipase solution was added to the substrate suspension, and incubation was performed in a controlled temperature bathtub (37°C) for up to 30 min.

### Characterization of the Lido-PPIX@ER hydrogel

For SEM analysis, Lido-PPIX@ER, and Lido-PPIX@ER hydrogel samples were prepared as follows. The samples were treated with 2.5% (v/v) glutaraldehyde in PBS (0.1 M, pH 7.4) for 3 h at 4°C. Next, after washing with PBS, the samples were mixed with different ethanol solutions (60%–100%) for 15 min at room temperature. All the samples were coated with a 10 nm thick gold film using a sputter coater, and the coated samples were examined using a JSM-6700F microscope (JEOL, Japan) in the secondary scattering and backscattering electron modes at a 10 keV electron acceleration voltage.

FITC-labeled lidocaine was tested *in vitro*. Images of ER-encapsulated FITC-Lido (green) and PPIX (red) were obtained using a Leica DM400 B LED (Leica, Germany) fluorescence microscope. LasX and Huygens were used for microscopy operation and data analysis, respectively.

The Lido-PPIX@ER was mixed with the amino acid hydrogel under stirring for 30 s using a vortex mixer, and then PFL was added. Photographs were taken at 2 and 8 min. The rheology experiment was performed using a rheometer at 37°C to determine the elastic modulus (G’) of the formed gel. The inflammatory activity of the Lido-PPIX@ER hydrogel was evaluated as follows: 1.0 ml of Lido-PPIX@ER hydrogel was subcutaneously injected into the back of the rat, and blocks of skin tissue from the injection site were collected on the third day and sliced. The anti-inflammatory activity was evaluated by imaging macrophages stained with rabbit anti-F4-80 fluorescent antibody. The sections were analyzed using a Leica SP8 STED instrument.

The weight fraction in percent vs. time and volume change of peptide hydrogel was tested as follows: After freeze-drying, the hydrogel was immediately weighed (W_0_), the volume was measured (V_0_), and the water absorption capacity of the samples was obtained. The wet specimens were weighed (W) and the volume (V) was measured at specified intervals after immersion in PBS at room temperature. The weight change was calculated as follows: weight fraction % = W/W_0_ × 100%. The volume change was calculated as follows: volume fraction % = V/V_0_ × 100%.

### *In Vitro* Release Behavior of the Lido-PPIX@ER hydrogel

The lidocaine concentration was determined by LC-2030 series HPLC (Shimadzu Corporation, Japan). The analysis was performed using a Hypersil OD S2 C18 5 µm column (250 × 4.6 mm, Å). The mobile phase was methanol/ammonium dihydrogen phosphate 0.01 M (NH_4_H_2_PO_4_) (25:75, v/v). The flow rate was set to 1.0 ml/min, and the detection was monitored at 220 nm.

The release behavior of the Lido-PPIX@ER hydrogel under different ultrasound powers (0–1 W/cm^2^ for 60 s) was monitored. The lidocaine concentration was monitored because the Lido@ER hydrogel (without PPIX) and Lido-PPIX@ER hydrogel (with 10 mg/ml of lidocaine in the ER) were triggered by ultrasound (0.3 W/cm^2^) for approximately 180 s. The spatiotemporally controlled release behavior of the Lido-PPIX@ER hydrogel was examined as 0.3 W/cm^2^ for repeated 30 s irradiation, and the assay was replicated a minimum of five times. HPLC was used to measure the PPIX concentration in the supernatant.

### Singlet Oxygen and MDA Determination

To analyze the laser irradiation-dependent release mechanism of the Lido-PPIX@ER hydrogel, singlet oxygen was labeled using a chemical probe (DCFH-DA; BestBio, Shanghai, China). The release at different time points was monitored based on fluorescence using a Leica DM400 B LED fluorescence microscope.

The malondialdehyde (MDA) concentration was measured using an MDA kit. The OD values of the measuring tube (ODm), control tube (ODc), standard tube (ODs) and blank tube (ODb) were measured at 533 nm.

The MDA concentration of Lido-PPIX@ER at different glucose concentrations was calculated as follows:

$$\text{MDA }\left(\text{nmol}/\text{L}\right)=\frac{\text{ODm}-\text{ODc}}{\text{ODs}-\text{ODb}}\times \frac{\text{standard substance concentration}}{\text{protein concentration}}$$

### Postoperative Pain Model

The surgery was performed according to the procedure described by Brennan et al. (1996). In this model, a 1 cm incision was made on the plantar surface of the left hind paw under isoflurane anesthesia (2% isoflurane in 100% oxygen). The incision was started immediately beginning at the distal end of the heel and extended to the proximal end of the first set of footpads. Forceps were used to elevate the plantar muscle, and the incision was lengthwise. The wound was sutured with two mattress sutures using 5-0 silk. After surgery, the rats recovered from anesthesia in their cages. Wounds were checked for signs of dehiscence before the behavioral test.

### *In Vivo* Behavioral Testing

To assess the *in vivo* management of postoperative pain, Lido-PPIX@ER hydrogel was subcutaneously injected around the created wound in the postoperative pain model rats before solidifying. The rats were divided into two groups (*n* = 10 per group)—namely, the free Lido group (6 mg) and 1 ml Lido-PPIX@ER hydrogel (with 5.6 mg lidocaine) + ultrasound group (0.3 W/cm^2^, 30 s).

The rats were placed in individual plastic chambers with a plastic mesh floor and allowed to acclimate to the environment for 30 min. The mechanical withdrawal threshold was measured using calibrated von Frey filaments (Stoelting, Wood Dale, IL, United States). The filaments were applied vertically in the area adjacent to the wound for 5–6 s with slight bending of the filament. Withdrawal of the hind paw from the stimulus was scored as a positive response. As described previously, the up-down method was used to identify tactile stimuli with a 50% likelihood of producing a withdrawal threshold (Chaplan et al., 1994).

Long-term postoperative pain management was administered as 30 s ultrasound irradiation (0.3 W/cm^2^, 30 s) every 2 h within 12 h after surgery, every 4 h from 12 to 48 h, and every 6 h from 48 to 72 h after surgery. The withdrawal threshold was determined after ultrasound triggering.

### Histological Observation

For histological evaluations, the postoperative pain model, treated with Lido-PPIX@ER hydrogel + ultrasound, was sacrificed to collect the skin tissue at the injection site and key organs, including heart, liver, kidney, lung, and spleen.

### Blood Biochemistry Index

Following the administration of Lido-PPIX@ER hydrogel + ultrasound for 5 and 15 days, an automated biochemical analyzer (Trilogy, France) was used to determine liver function indices, including alkaline phosphatase (ALP), alanine aminotransferase (ALT), and aspartate aminotransferase (AST). Likewise, the kidney function markers blood urea nitrogen (BUN), creatinine (Cr), and globulin (GLB) were also evaluated (Wang et al., 2019).

### Statistical Analysis

All values were reported as means ± SD unless stated otherwise. Statistical analysis was performed using SPSS 18.0 software. Multiple variables were compared using ANOVA, while two groups were compared using two-sample *t*-test. *p* < 0.05 was considered statistically significant.

## Results

### Preparation of Lido-PPIX@ER

**FIGURE 1:**
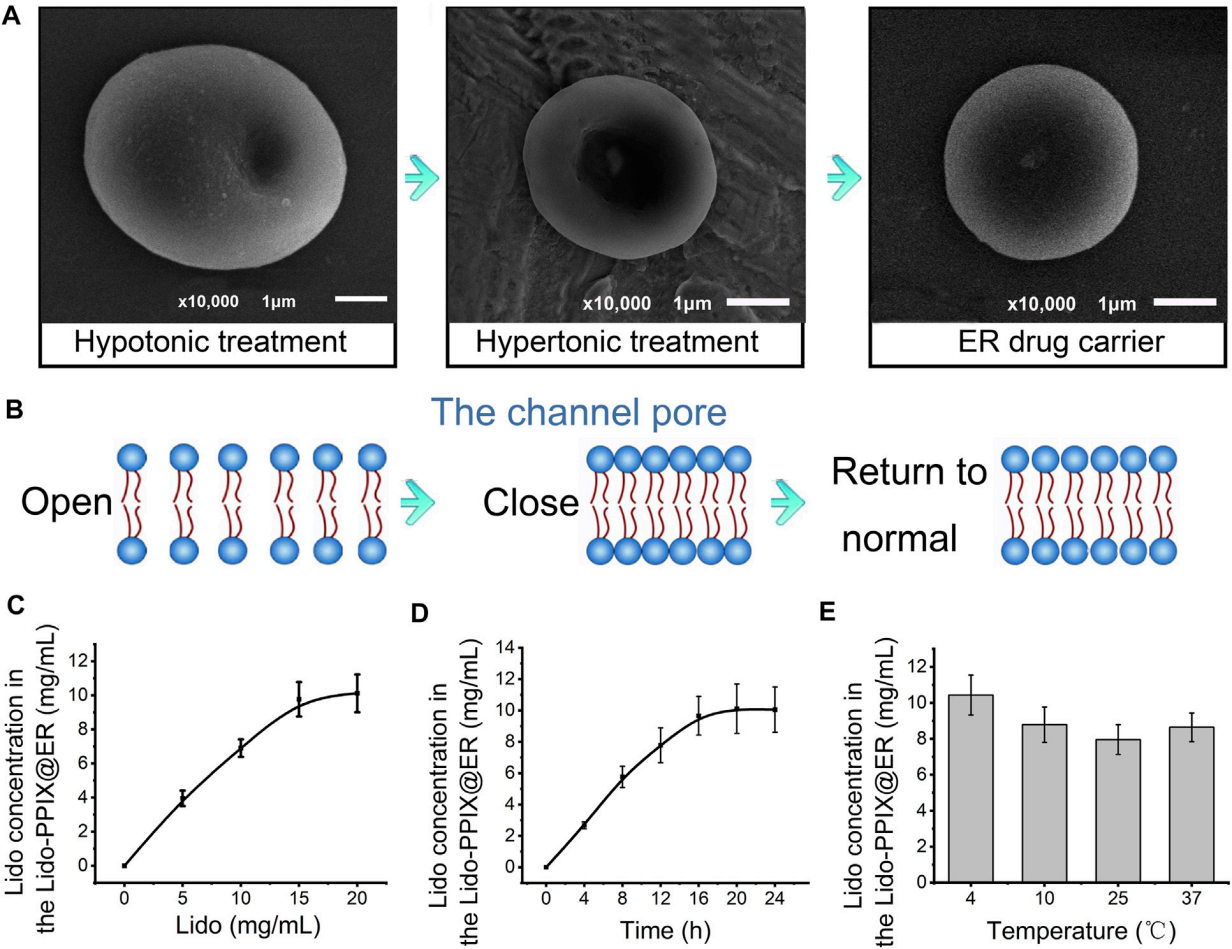
Construction of Lido-PPIX@ER. **(A)** SEM images of Lido-PPIX@ER during hypotonic, hypertonic or PBS treatment. **(B)** Schematic diagram of phospholipid bilayer changes during hypotonic, hypertonic or PBS treatment. **(C)** Relationship between the initial Lido concentration and loading content. **(D)** Effect of dialysis duration on the Lido loading content. **(E)** Relationship between the temperature and loading content.

In the present study, ERs were used as lidocaine carriers because of their high drug loading capacities and long *in vivo* circulation features in vessels (Xia et al., 2018). The lidocaine content inside Lido-PPIX@ER is a key factor that affects the therapeutic efficiency in postoperative pain. During the preparation of Lido-PPIX@ER, the ERs were first dispersed in a hypotonic solution to achieve a pre-swelling status to facilitate drug loading (Figure 1A). The ER carriers bulged and became enlarged, but maintained their original shape (concave disk-like). Subsequently, the Lido-PPIX@ER was dispersed in a hypertonic solution for reannealing. The ER carriers became smaller, and the fovea of the ERs became enlarged. Finally, the Lido-PPIX@ER was dispersed in isotonic solution for resealing. Because the ER cell membrane comprises phospholipid bilayers, the high tension (hypoosmotic conditions) loosened the bilayers and allowed the drugs to enter (Figure 1B). Similarly, the bilayers became compact under hypertonic reannealing and returned to normal under isotonic resealing. Thus, we established a practical lidocaine-loaded ER system.

To achieve a practical lidocaine-loaded erythrocyte system, the optimal loading condition was first challenged using a procedure involving the lidocaine concentration, dialysis time and temperature. The loading content of lidocaine increased with increasing Lido concentration until it approached 15 mg/ml (Figure 1C). Extending the incubation time also resulted in more Lido entering ERs to reach equilibrium (Figure 1D). The temperature of 4°C was selected as the best reaction temperature in hypotonic dialysis (Figure 1E).

**FIGURE 2:**
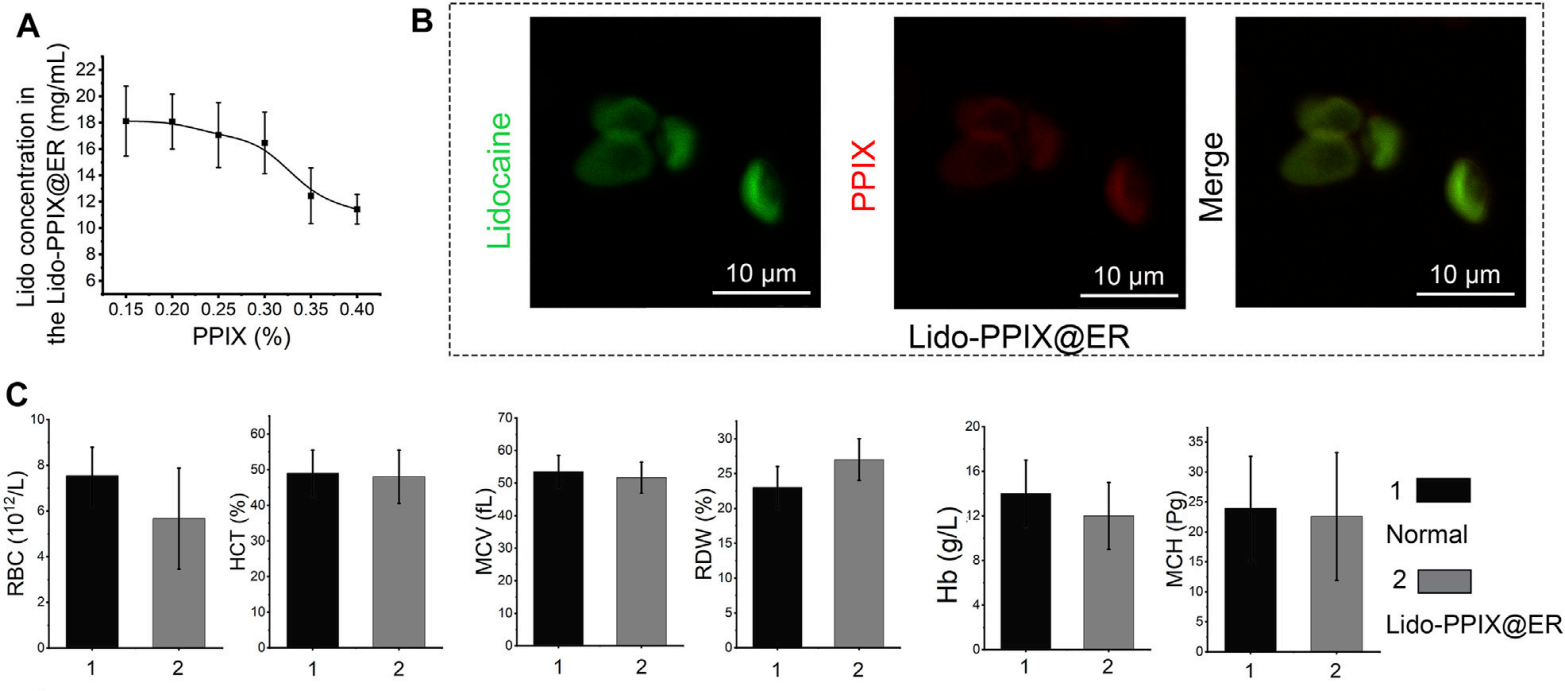
Structure of Lido-PPIX@ER. **(A)** Relationship between the initial PPIX concentration and loading content. **(B)** Confocal images of FITC-labeled Lido (green), PPIX (red), and merged fluorescence. **(C)** Hematological parameters of normal blood and Lido-PPIX@ER, including red blood cells (RBCs), hematocrit (HCT), mean corpuscular volume (MCV), red cell distribution width (RDW), hemoglobin (Hb), and mean corpuscular hemoglobin (MCH).

In the present study, PPIX was loaded inside the ER to act as an ultrasound-activated switch to control lidocaine release. The resulting decrease in the lidocaine concentration in ERs (Figure 2A) indicated that PPIX and ER bind competitively to lidocaine. An optimized PPIX concentration (0.3%) was adopted to achieve a high lidocaine loading content without compromising the Lido-loading efficiency. To further demonstrate that lidocaine and PPIX were enclosed within ERs, the loading of lidocaine and PPIX was characterized by fluorescence imaging (Figure 2B). FITC-labeled PPIX (green) and lidocaine (red) were both observed in ERs. The overlapping image of Lido and PPIX demonstrated the successful encapsulation of lidocaine and PPIX inside Lido-PPIX@ER.

Even after the loading of lidocaine and PPIX, ERs maintained their original shape (concave disk-like) and size. Additionally, hematological parameters showed no significant differences between the native ER and Lido-PPIX@ER, such as the RBC number (RBC), hematocrit (HCT), mean corpuscular volume (MCV), red blood cell distribution width (RDW), hemoglobin (Hb), and mean corpuscular hemoglobin (MCH) (Figure 2C). These results suggested that the encapsulation of Lido and PPIX did not alter the biological properties of the ERs. The stabilities of Lido-PPIX@ER were examined as the size, morphology, and stability in PBS or culture medium (Supplementary Figures S1, S2). These results demonstrated that the Lido-PPIX@ER were stable during storage, and provided a possible favorable condition for preparing in advance.

### Preparation of the Lido-PPIX@ER Hydrogel

**FIGURE 3:**
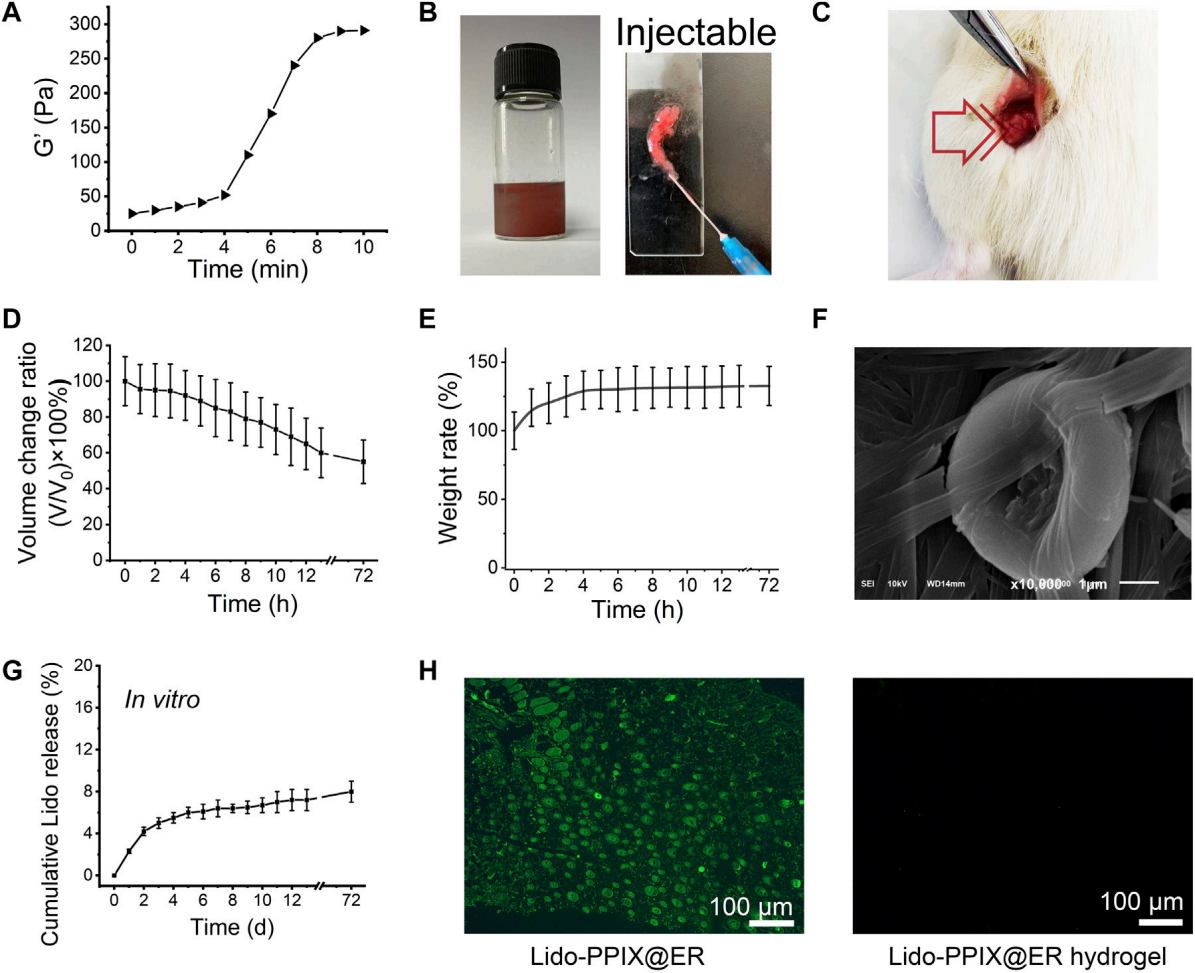
Characterization of the Lido-PPIX@ER hydrogel. **(A)** Rheology properties of PFL-catalyzed Fmoc-Phe_3_. **(B)** Photographs showing the gelation behavior of the Lido-PPIX@ER hydrogel after adding PFL for 2 and 10 min and photograph of the injectable Lido-PPIX@ER hydrogel. **(C)** Photograph of peptide hydrogel *in vivo*. The peptide hydrogel was subcutaneously injected within 2 min. Ten minutes later, the mixture and PFL could also shift from liquid to a homogeneous gel state *in vivo*. **(D)** Volume change curve of the Lido-PPIX@ER hydrogel in PBS at 37°C. **(E)** Weight fraction in precent vs. time of Lido-PPIX@ER hydrogel in PBS at 37°C. **(F)** The SEM imge of Lido-PPIX@ER hydrogel. **(G)** Stability of Lido-PPIX@ER hydrogel *in vitro*. **(H)** Immunohistochemical staining for f4/80 to identify infiltrated macrophages in the subcutaneous tissues. The experiment was repeated at least three times.

The ER drug carriers were administered subcutaneously and scavenged by macrophages (Millán et al., 2004). To extend their lifetime *in vivo*, the Lido-PPIX@ER were embedded in a stable scaffold comprising an amino acid hydrogel, which could be subcutaneously implanted. *Pseudomonas fluorescens* (PFL) was used to catalyze the formation of a peptide bond between Fmoc-Phe and Phe2 at 37°C. The mechanical properties of this hydrogel obtained at different reaction times are shown in Figure 3A and Supplementary Figure S2A. After 8 min, the highest rheological property was reached and maintained a constant value for at least 4 days. The effect of PLF on the mechanical of hydrogel cured was investigated (Supplemantary Figure S2B). We observed a rapid rise in compressive strength of hydrogel and it was maintained on subsequent days. The lipase-catalyzed reaction rapidly changed the reaction medium from solution to gel, enhancing the mechanical properties. When the reaction was completed, the hydrogel was no longer formed, resulting in a constant mechanical property.

Mixing Lido-PPIX@ER and amino acids (1:1, vol/vol) formed a cohesive network (Lido-PPIX@ER hydrogel) following the addition of lipase (PFL). After lipase addition, the mixture of Lido-PPIX@ER and PFL shifted from liquid to a homogeneous gel state (Figure 3B). Next, the changes from liquid to gel *in vivo* were examined. The peptide hydrogel was subcutaneously injected within 2 min. Ten minutes later, the mixture and PFL also shifted from liquid to a homogeneous gel state *in vivo* (Figure 3C).

The hydrogel worked as a scaffold to support the Lido-PPIX@ER and prolonged the action time. We hypothesized that the hydrogel could support the structure of Lido-PPIX@ER because a small change in the volume of the Lido-PPIX@ER hydrogel was observed during the following 3 days (Figure 3D). The hydrogel swelled in PBS, resulting in a slight increase in weight in the first 6 h. Next, it reached weight balance and maintained a constant weight for more than 3 days (Figure 3E).

This transforming ability ensured that the Lido-PPIX@ER hydrogel was injected into a mold to obtain a stable 3D structure. The SEM image in Figure 3F illustrates the porous structure of this obtained amino acid hydrogel comprising a 3D network with microchannels. ERs, exhibiting a typical concave disk structure similar to normal ERs, were still clearly observed in this hydrogel. As envisioned, the Lido in the Lido-PPIX@ER hydrogel exhibited excellent stability *in vitro* because most of the Lido still exited the ERs (Figure 3G). Combining the results in Figure 1, we confirm the successful loading of ERs.

Good compatibility, without an inflammatory-like response, is a vital feature of subcutaneous scaffolds to prevent macrophage infiltration, preventing the early destruction of drug-loaded ER (Rose and Currow, 2009). The infiltrated macrophages in the subcutaneous tissues after the hydrogel implant were labeled with f4/80 (green) and are shown in Figure 3H. Compared with the direct injection of Lido-PPIX@ER, the injection of Lido-PPIX@ER hydrogel did not induce significant infiltration of macrophages even after 5 days. This finding partly demonstrated that the Lido-PPIX@ER hydrogel would not induce inflammation *in vivo*.

### Ultrasound-Triggered *In Vitro* Release of Lido From Lido-PPIX@ER Hydrogel

**FIGURE 4:**
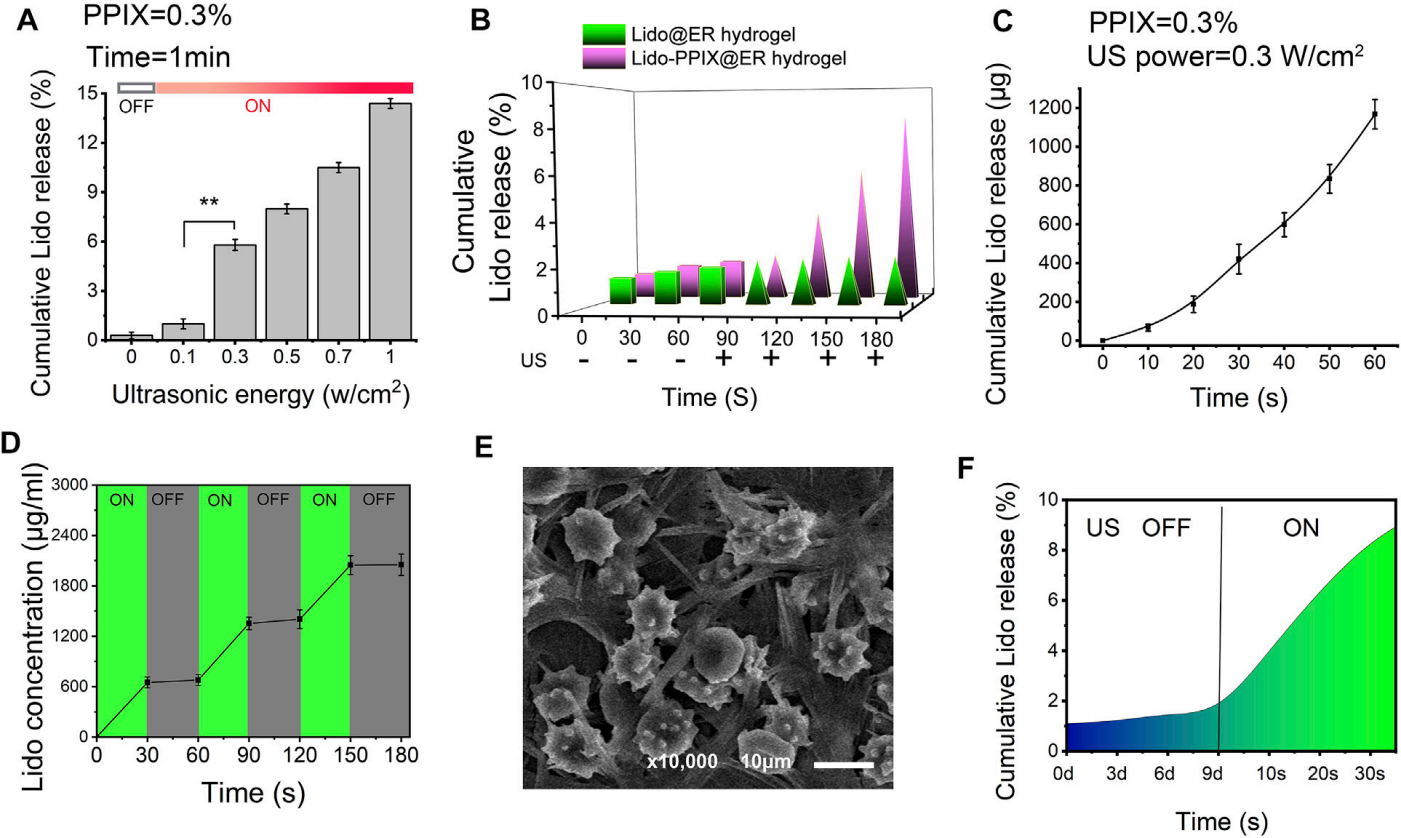
Ultrasound-triggered release behavior of the Lido-PPIX@ER hydrogel. **(A)** *In vitro* lidocaine release from the Lido-PPIX@ER hydrogel at increasing ultrasound power at 37°C. **(B)** *In vitro* lidocaine release from the Lido@ER hydrogel or Lido-PPIX@ER hydrogel with or without laser intensities at 37°C (0.3 W/cm^2^, 60 s). **(C)** Release profile of lidocaine from 1.0 ml of Lido-PPIX@ER hydrogel during continuous ultrasound triggering. **(D)** *In vitro* release profile of lidocaine when the ultrasound was cyclically varied between on and off for several repetitions. **(E)** Morphological changes of Lido-PPIX@ER during “on-off” lidocaine release. **(F)** Ultrasound-triggered release behavior of the Lido-PPIX@ER hydrogel after storage in PBS at 4°C for 9 days.

We expected that when the Lido-PPIX@ER hydrogel is triggered by ultrasound, PPIX will produce ROS, which consequently destroy the integrity of the ER membrane, leading to Lido release. To confirm the ultrasound-triggerable response leakage of Lido, the release of Lido from the Lido-PPIX@ER hydrogel was monitored by ultrasound from 0 to 1 W/cm^2^ for 60 s (Figure 4A). The burst release of Lido was observed when the ultrasound power was greater than 0.3 W/cm^2^ (*p* > 0.01), while almost no Lido was released when the power was off. This result demonstrated that ultrasound (0.3 W/cm^2^) was effective in regulating Lido release from the Lido-PPIX@ER hydrogel.

By contrast, almost no Lido was released from the Lido@ER hydrogel (without PPIX) with/without ultrasound or the Lido-PPIX@ER hydrogel without ultrasound (Figure 4B). Interestingly, Lido release from the Lido-PPIX@ER hydrogel could be accelerated with the extension of ultrasound (Figure 4C). To evaluate the responsible release behavior of the Lido-PPIX@ER hydrogel, the Lido concentration was monitored when the ultrasound was cyclically turned on and off every 30 s. Lido released from the 1 ml Lido-PPIX@ER hydrogel responded to 0.3 W/cm^2^ ultrasound irradiation for 30 s (Figure 4D). The concentration of lidocaine increased as the ultrasound was turned on, while lidocaine was no longer released from the Lido-PPIX@ER hydrogel when the power was turned off. The effect of ultrasound on the morphology change of Lido-PPIX@ER was examined by SEM (Figure 4E). With the power on, the Lido-PPIX@ER showed an irregular morphology, and morphological changes of ER were observed facilitating lidocaine release.

Unsurprisingly, the Lido-PPIX@ER hydrogel maintained its bioactivity even after 9 days in PBS, representing a crucial property of Lido-PPIX@ER for postoperative pain management (Figure 4F). The Lido concentration in the supernatant was very low in the first 9 days, indicating that most of the Lido was stored inside the ERs and would not be released into PBS. On Day 9, the remaining Lido-PPIX@ER hydrogel was treated with ultrasound. The Lido concentration in the suspension increased rapidly after 30 s.

### Releasing Mechanism of the Lido-PPIX@ER Hydrogel

**FIGURE 5:**
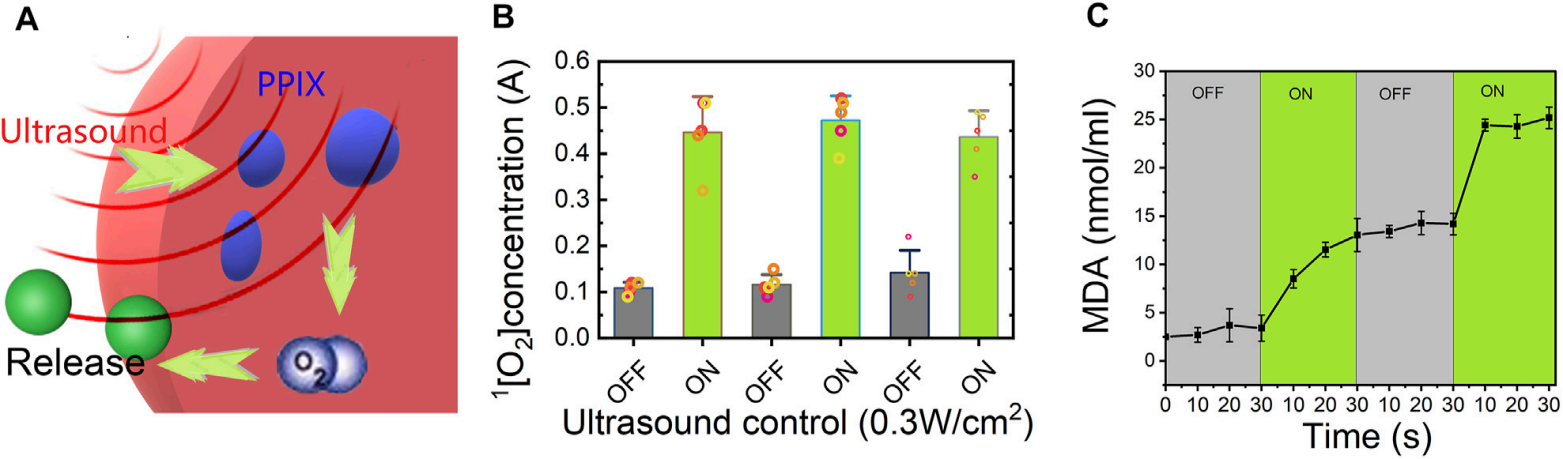
Release mechanism of the Lido-PPIX@ER hydrogel under ultrasound. **(A)** Schematic representation showing the ultrasound-regulated release of lidocaine from the Lido-PPIX@ER hydrogel. **(B)** Concentration profile of singlet oxygen with ultrasound (0.3 W/cm^2^) in the on/off state. **(C)** MDA concentration changes of the Lido-PPIX@ER hydrogel under ultrasound regulation (on-off state) at 37°C.

We expected that when the Lido-PPIX@ER hydrogel is irradiated by ultrasound, PPIX will produce ROS, which consequently destroy the integrity of the ER membrane, leading to Lido release. To confirm the ultrasound-triggered leakage of Lido, Lido release from the Lido-PPIX@ER hydrogel was monitored following ultrasound treatment (0.3 W/cm^2^, 30 s) (Figure 5A). Given that the lipid bilayer membrane of ERs would be damaged in the presence of accumulated ROS to release the packed drugs (Xia et al., 2018), the relationship between ultrasound treatment and ROS production was studied and is shown in Figure 5B.

The ROS concentration was directly related to ultrasound because PPIX generated singlet oxygen after triggering. Once the ultrasound was turned off, ROS were no longer produced, and the residual ROS could be scavenged by SOD and CAT, leading to ROS exhaustion in the hydrogel. Therefore, the ROS concentration showed pulsate changes with the laser on and off. The accumulated ROS promoted pore formation in the ER membrane, allowing Lido release. Likewise, ROS generation was stopped when ultrasound was turned off, leading to closure of the pores in the ER membrane and inhibited PPIX release.

Additionally, we evaluated changes in the malondialdehyde (MDA) levels in Lido-PPIX@ER as an indicator of oxidative stress (Figure 5C). The changes in the MDA and H_2_O_2_ levels were consistent, reflecting ultrasound triggering.

### Postoperative Pain Management *In Vivo*

**FIGURE 6:**
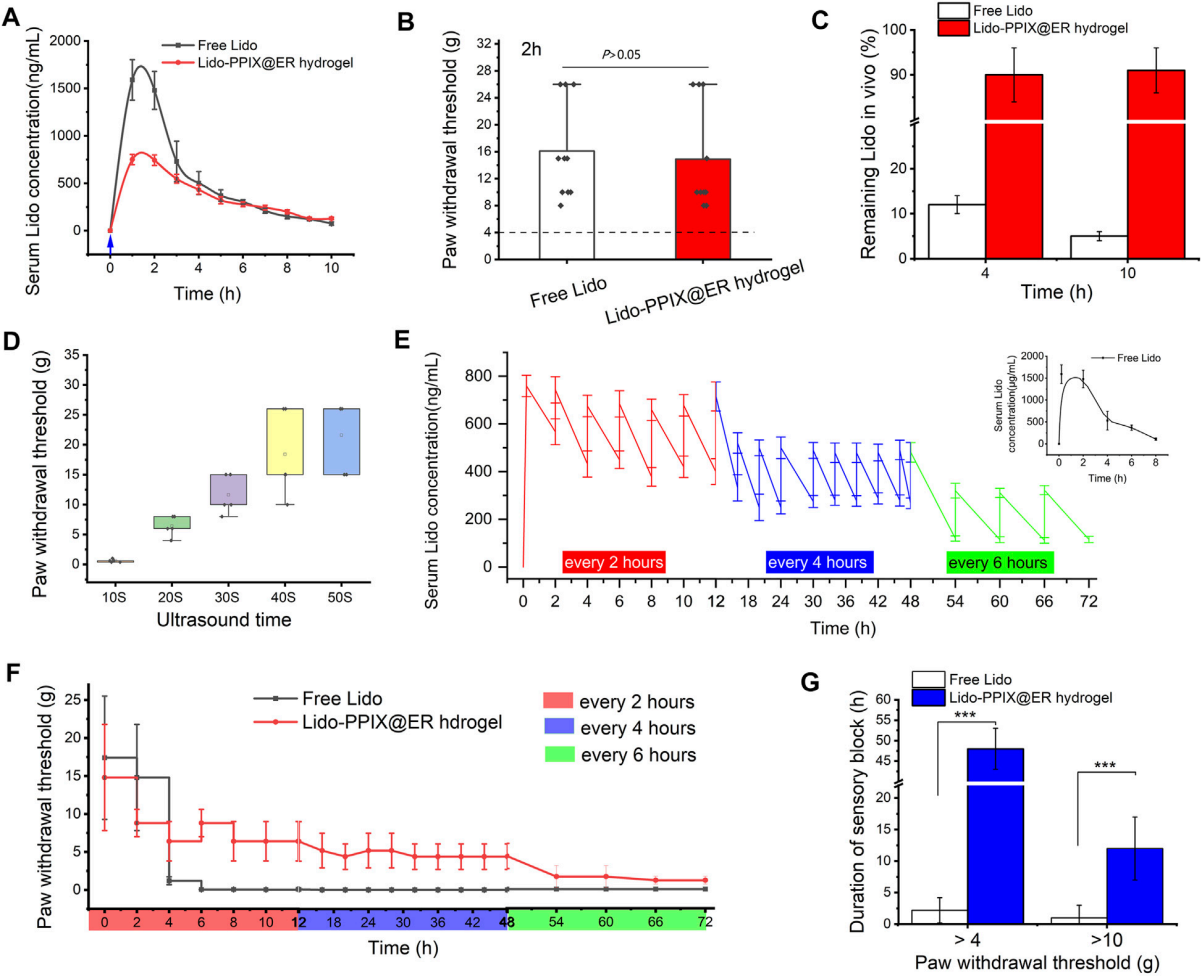
Effects of managing postoperative pain *in vivo*. **(A)** Change curve of the serum Lido concentration and **(B)** paw withdrawal threshold (2 h after surgery) after one ultrasound trigger. **(C)** Ratio of the remaining lidocaine in free lidocaine and Lido-PPIX@ER hydrogel after one ultrasound-triggered removal from the administration site in rats. **(D)** Duration of ultrasound-triggered lidocaine release and effect of pain relief application in the rat model of postoperative pain in the Lido-PPIX@ER hydrogel-treated group. **(E)** Change curve of the serum Lido concentration and **(F)** the hind paw withdrawal thresholds after on-demand ultrasound triggering in the Lido-PPIX@ER hydrogel group (triggered for every 2 h in the first 12 h and every 4 h for the next 36 h, and then every 6 h until 72 h after surgery). **(G)** Effective time at different paw withdrawal thresholds in the ultrasound-triggered Lido-PPIX@ER hydrogel group. ***, *p* < 0.001.

To investigate the possibility of the Lido-PPIX@ER hydrogel controlling postoperative pain with ultrasound triggering, we treated a postoperative pain rat model with the Lido-PPIX@ER hydrogel. First, the mean serum Lido concentration after free Lido (6 mg) or 1 ml of Lido-PPIX@ER hydrogel (containing 5.6 mg/ml of lidocaine) treatment must be evaluated. In the lidocaine (6 mg)-treated group, the mean serum lidocaine concentration steadily increased to 1,790 ng/ml at 2 h after subcutaneous administration (Figure 6A). In the Lido-PPIX@ER hydrogel-treated group, the serum lidocaine concentration did not show a burst increase after ultrasound triggering (0.3 W/cm^2^, 30 s).

To further prove that the ultrasound-triggered Lido-PPIX@ER hydrogel could be used in postoperative pain management, mechanical hypersensitivity was observed 2 h after paw incision (Figure 6B). Subcutaneous administration of the Lido-PPIX@ER hydrogel produced antihypersensitivity effects compared with the control group (*p* > 0.05). We confirmed that the ultrasound course of lidocaine released by the Lido-PPIX@ER hydrogel *in vivo* was similar to that of free Lido to manage postoperative pain in the first 4 h.

As expected, examination of the residual Lido-PPIX@ER hydrogel removed from rats after one ultrasound-triggered (0.3 W/cm^2^, 30 s) *in vivo* treatment revealed that approximately 91% and 90% of the administered lidocaine remained at 4 and 10 h, respectively (Figure 6C). Conversely, approximately 11 and 6% of the administered lidocaine remained at 4 and 10 h, respectively, in the free Lido-treated group. Thus, sufficient subcutaneous storage of the Lido-PPIX@ER hydrogel provides a favorable condition for long-term ultrasound-triggered postoperative pain management.

The on-demand release of Lido in postoperative pain management meets the needs of the most acute care. We next tested the on-demand release behavior of the Lido-PPIX@ER hydrogel under different ultrasound-triggered times. With increasing ultrasound-triggered time, the Lido concentration increased as expected (Figure 6D).

Ultrasound at 0.3 W/cm^2^ for 30 s for triggered release in the Lido-PPIX@ER hydrogel was optimal because it achieved a stable plasma concentration (Figure 6E). After surgery, severe pain continued for approximately 12 h and was relieved. To address this issue, ultrasound was triggered every 2 h in the first 12 h, every 4 h for the next 36 h, and then every 6 h until 72 h after surgery. Thus, the mean serum lidocaine concentration was steadily high initially and then decreased from 592 ng/ml at 12 h to 487 ng/ml at 18 h after the administration of the ultrasound-triggered time interval. Unsurprisingly, the mean serum lidocaine concentration from 48 to 72 h after surgery decreased to a lower level. Naked Lido without protection, by contrast, can be cleared from organisms by 4–8 h after subcutaneous administration.

The paw withdrawal threshold changed with the mean serum lidocaine concentration (Figure 6F). The group treated perineurally with repeatedly ultrasound-triggered Lido-PPIX@ER hydrogel showed stable withdrawal thresholds at various time periods, higher concentrations at all time points after paw incision than the free Lido-treated group, and lasted approximately 72 h. However, the free lidocaine treatment group disappeared 4 h after paw incision, and the paw withdrawal thresholds were decreased to less than 4 g.

To better fit the needs of men with different pain thresholds, the effective time at different paw withdrawal thresholds was examined (Figure 6G). The Lido-PPIX@ER hydrogel controlled the paw withdrawal thresholds over 4 g or 10 g for approximately 48 and 12 h compared with the free lidocaine-treated group (*p* < 0.001). Therefore, repeated administration of the Lido-PPIX@ER hydrogel meets the requirement to ensure sustained postoperative pain management.

### Safety Analysis

**FIGURE 7:**
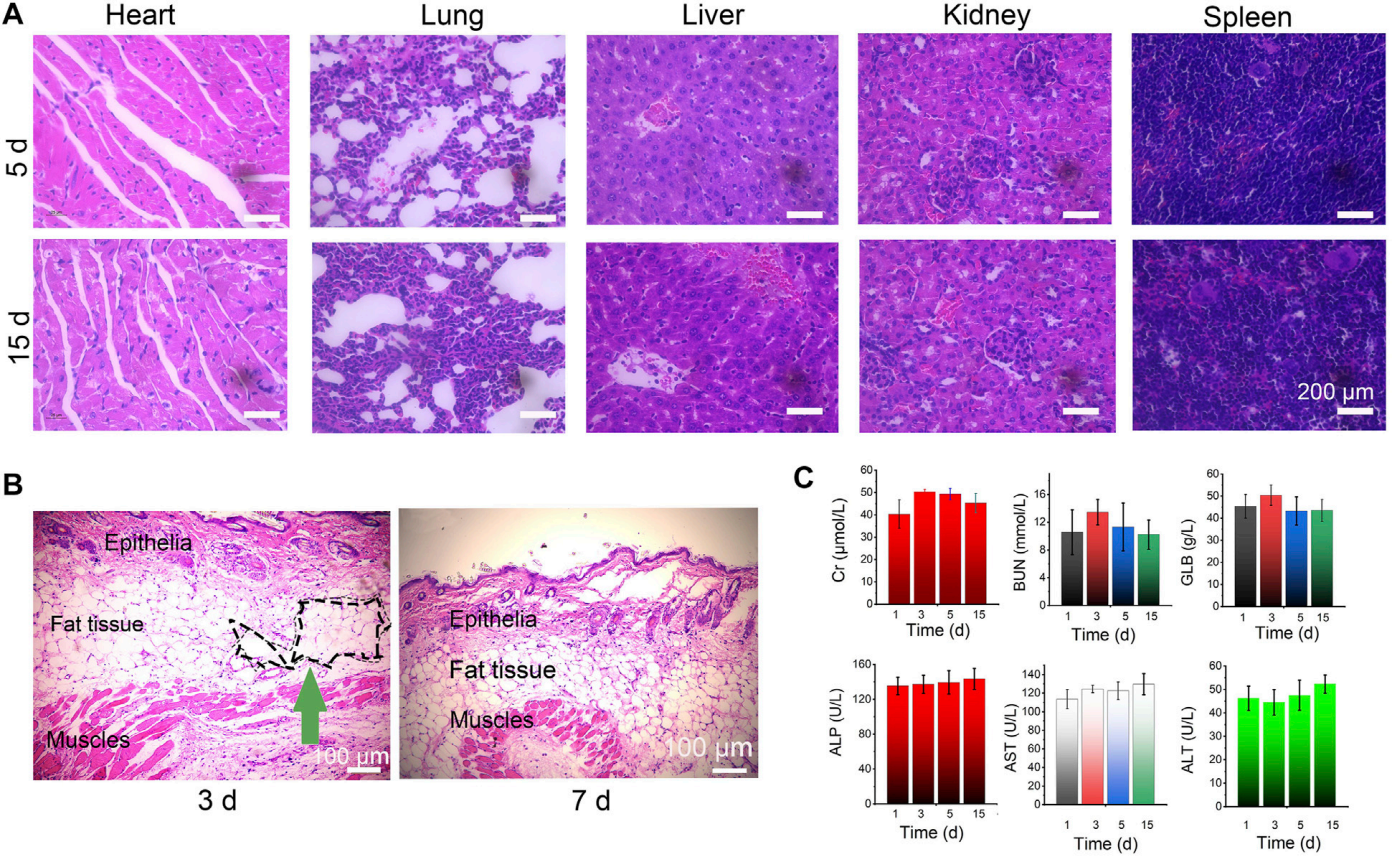
Biosafety of the Lido-PPIX@ER hydrogel *in vivo*. **(A)** Images of H&E staining after treatment with the Lido-PPIX@ER hydrogel. **(B)** H&E-stained sections of surrounding tissues at the site of subcutaneously injected Lido-PPIX@ER hydrogel. Scale bar = 100 µm. **(C)** Kidney function (BUN, Cr, and GLB) and the levels of liver (ALP, ALT, and AST) markers on Days 1, 3, 5, and 15 in the control and Lido-PPIX@ER hydrogel treatment groups.

Next, to evaluate the potential health risks after treatment with the Lido-PPIX@ER hydrogel plus laser irradiation, the treated mice were euthanized on Day 5 and 15. The main organs, including the heart, liver, kidney, lung, and spleen tissues, were stained with hematoxylin and eosin (H&E) (Figure 7A).

To evaluate the *in vivo* degradability of the Lido-PPIX@ER hydrogel, the size of the resultant subcutaneous skin lump was monitored over time (not shown). We noticed that 15 days post-injection, no obvious skin protrusion was noticed, illustrating the complete degradation of the Lido-PPIX@ER hydrogel. From the H&E staining results of surrounding tissues at the site of subcutaneously injected Lido-PPIX@ER hydrogel, the lesion region was covered with connective tissue after 15 days (Figure 7B). Collectively, the hydrogel provided structural support and exhibited self-degradation *in vivo*.

Furthermore, analysis of liver function (ALP, ALT, and AST) and kidney function (BUN, Cr, and GLB) markers (Figure 7C) revealed that Lido-PPIX@ER hydrogel + ultrasound treatment did not cause hepatic or kidney damage. Therefore, the Lido-PPIX@ER hydrogel exhibits good biocompatibility *in vivo*.

## Discussion

To maximize postoperative pain relief, enhance patient satisfaction, and facilitate postoperative rehabilitation, pain management is essential. Lidocaine has been used in clinical medicine for more than half a century, and multimodal lidocaine-containing products have proven effective to treat postoperative pain. However, lidocaine may induce nerve demyelination and contribute to the neurotoxicity of lidocaine, and multiple dosing delivery was used in the clinic using conventional intravenous administration. This presents a challenge, such as the requirement of skilled professionals, a situation that is not easily achievable for families. Studies specifically designed to address this issue are required. Sustained-release local anesthetics have been reported to treat postoperative pain (Wang et al., 2016). The liposomal bupivacaine injectable suspension (EXPAREL^®^; Pacira Pharmaceuticals, Inc., United States) has received FDA Approval for infiltration into a surgical site to provide postoperative analgesia in adults based on clinical trials in which subjects had undergone bunion reaction (Golf et al., 2011) and hemorrhoidectomy (Gorfine et al., 2011), demonstrating efficacy for 36 and 72 h, respectively. Furthermore, clinical trials of liposomal bupivacaine for epidural block (Viscusi et al., 2012), femoral nerve block (Surdam et al., 2015), transversus abdominis plane block (Fayezizadeh et al., 2016) and interscalene block (Namdari et al., 2017) have been reported previously. However, different people have different pain thresholds or pain tolerance scores. Therefore, an effective treatment for postoperative pain should consider variable timing and intensity of anesthetics.

Generally, men show severe pain on the day after the operation. With the extension of the treatment time, the postoperative pain is reduced over time. Different people have different pain thresholds or pain tolerance scores. Relieving local pain as needed, allowing patients to control the timing and intensity, would be the excellent choice to maximize personalized postoperative pain. An ultrasound-triggerable, on-demand nerve block system allows control of the duration and intensity of nerve block simply using the duration and intensity of the ultrasound (Rwei et al., 2017). In the present study, ultrasound-triggered on-demand local anesthesia was reported, and the intensity and duration of local anesthesia were controlled by adjusting the intensity and duration of the ultrasound. The Lido-PPIX@ER hydrogel described here allowed an on-demand release of lidocaine from the subcutaneous lidocaine reservoir by remote ultrasound control without frequent injection. Thus, additional time for on-demand nerve blocks is allowed, leading to personalized anesthesia-free pain management.

Alternatively, a sufficient action time must be considered in its clinical application because patients undergoing major spine surgery frequently experience severe postoperative pain lasting more than 3 days. Here, we designed a large capacity lidocaine vector that controls the timing, intensity and duration of lidocaine to relieve postoperative pain. Recently, the erythrocyte membrane has been extensively studied to develop novel drug delivery systems to prolong the cyclic persistence of drugs. As “innate carriers,” ER have many unique characteristics, such as perfect biocompatibility, a long circulation half-life (approximately 120 days in humans), membrane flexibility and stability. In the present study, ER were used as lidocaine carriers because of their high drug loading capacities and long *in vivo* circulation features in vessels (Xia et al., 2018). Furthermore, the presence of accumulated H_2_O_2_ caused the pores in the erythrocyte membrane to open rapidly. Considering that PPIX produces H_2_O_2_ when triggered by ultrasound, we describe the development of an ultrasound-triggered drug release platform (Lido-PPIX@ER hydrogel) for postoperative pain management. From the results in Figure 3, the drug release platform was very stable *in vivo*, providing a prerequisite to obtain ultrasound-triggered on-demand release behavior.

Ultrasound, as an integral part of drug-delivery modalities, has many attractive features, including simplicity and cost-effectiveness (Covotta et al., 2020). Sonication can be performed non-invasively, and ultrasound waves can be directed to deep locations for precise energy-deposition patterns (de la Fuente et al., 2021; Mi et al., 2021). In early studies, microbubbles, which are clinically recognized as ultrasound contrast agents, were used to load therapeutic agents for effective tumor chemotherapy. However, the shortcomings of these gas-filled microbubbles prevent their widespread use in drug delivery. In the present study, the hypothesis that sonochemistry and ROS production played a major role in ultrasound-triggered cargo release is supported by the observation that PPIX-loaded ER produced more ROS, opening the ER phospholipid bilayer and triggering drug release. Ultrasound-induced cavitation can produce light (sonoluminescence), which activates the photosensitizer PPIX to produce ROS. Implosion of ultrasound-induced cavitation bubbles can also induce the formation of sonosensitizer-derived free radicals that generate ROS. These effects depend on inertial cavitation following insonation, which may be caused by the ultrasound conditions used here (0.3 W/cm^2^). Therefore, we designed a novel multimode osmotic analgesia scheme for wound infiltration.

In this study, we report an injectable, remote ultrasound-induced lidocaine delivery system for postoperative pain management. The Lido-PPIX@ER hydrogel exhibited high lidocaine loading capacity with efficient ultrasound-triggered release of lidocaine, making it a potential delivery system for well-regulated postoperative pain. *In vivo* experiments in a postoperative pain model showed that a single injection of Lido-PPIX@ER hydrogel can effectively control the duration and intensity of nerve block by simply adjusting the duration and intensity of ultrasound. Therefore, we suggest that this delivery system is an effective strategy to reduce pain, remotely control pain, and offer timed and spatiotemporally controlled release of Lido to manage postoperative pain. Although, this kind of ultrasound-triggered on demand release system cannot be applied clinic in present form due to limited source of ER, we hope this Lido delivery stratagem can provide new insights for the management of postoperative pain.

## Data Availability

The original contributions presented in the study are included in the article/Supplementary Material, further inquiries can be directed to the corresponding authors.
