# Supplementary material for: Ultrasound-Triggered on Demand Lidocaine Release Relieves Postoperative Pain
Source: Front Bioeng Biotechnol. 2022 Jul 11;10:925047. doi: 10.3389/fbioe.2022.925047 (PMC9310090; doi:10.3389/fbioe.2022.925047)
Supplement: Supplementary file 1 [file DataSheet1.docx]

Supplementary Material

Ultrasound-triggered on Demand Lidocaine Release Relieves Postoperative Pain

**Xiaohong Chen^1,2, †^, J****ianfeng Zhang^2, †^, Yan Yu^2^, Haoran Wang^2^, Genshan Ma^2^, Di Wang^2^, Hanzhong Cao^2*^,** **Jianping Yang^1*^**

^1^ The Frist Affiliated hospital of Soochow University, Suzhou, Jiangsu, China

^2^ Nantong Tumor Hospital, Tumor Hospital Affiliated to Nantong University, Nantong, Jiangsu, China

**^†^** These authors have contributed equally to this work and share first authorship

*** Correspondence:**Corresponding Author
[chz-zp@163.com](mailto:chz-zp@163.com) (H.C Cao), [YangJP1956@126.com](mailto:YangJP1956@126.com) (J.P Yang)

Keywords:

Ultrasound-triggered drug release, Postoperative pain, Lidocaine, On-demand release, Erythrocytes

## Supplementary Figures


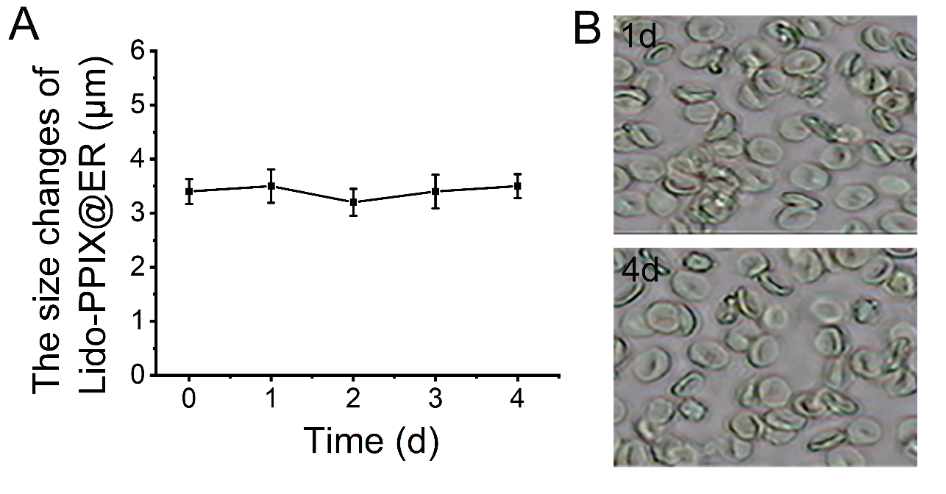


**Supplementary Figure 1.** The stability of Lido-PPIX@ER in PBS at 4 °C. (A) The size changes of Lido-PPIX@ER. The volumes Lido-PPIX@ER of remain stable at least 4 days. (B) The morphology of Lido-PPIX@ER on 1d and 4d. To understand the morphologic changes of Lido-PPIX@ER, erythrocytes after loading Lido and PPIX were observed using an optical microscope. The Lido-PPIX@ER maintained their typical biconcave disk shape and showed no visible change.


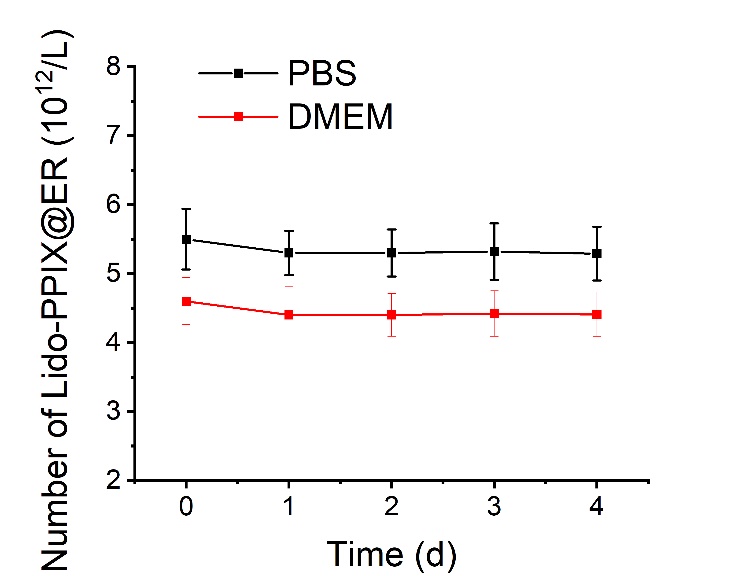


**Supplementary Figure 2.** The change in number of Lido-PPIX@ER in PBS and DMEM at 4 °C. No decrease in the number of Lido-PPIX@ER was observed, either in PBS or DMEM. It showed that the Lido-PPIX@ER was stable.


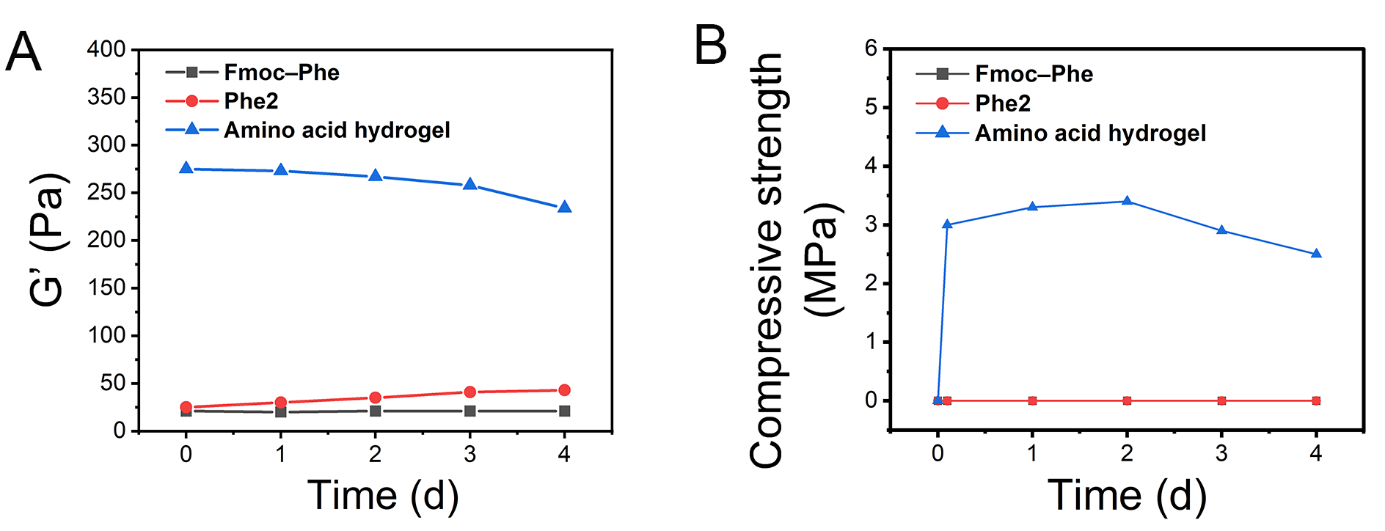


**Supplementary Figure 3.** The rheological (A) and mechanical (B) properties of hydrogel. After 8 min, the highest rheological property and compressive strength of hydrogel was reached and maintained a constant value for at least 4 days. Reciprocally, the rheological and mechanical properties Fmoc-Phe and Phe2 were close to 0.
